# Hypofractionated stereotactic re-irradiation for progressive glioblastoma: twelve years’ experience of a single center

**DOI:** 10.1007/s11060-024-04607-4

**Published:** 2024-02-22

**Authors:** Melek Tugce Yilmaz, Alper Kahvecioglu, Gozde Yazici, Sepideh Mohammadipour, Neyran Kertmen, Gokcen Coban Cifci, Faruk Zorlu

**Affiliations:** 1 Department of Radiation Oncology, Hacettepe University Faculty of Medicine, Ankara, Turkey; 2 Department of Medical Oncology, Hacettepe University Faculty of Medicine, Ankara, Turkey; 3 Radiology Department, Hacettepe University Faculty of Medicine, Ankara, Turkey

**Keywords:** Glioblastoma, Recurrence, Stereotactic radiotherapy, Hypofractionated radiosurgery

## Abstract

**Purpose:**

We aimed to evaluate the prognostic factors and the role of stereotactic radiotherapy (SRT) as a re-irradiation technique in the management of progressive glioblastoma.

**Methods:**

The records of 77 previously irradiated glioblastoma patients who progressed and received second course hypofractionated SRT (1–5 fractions) between 2009 and 2022 in our department were evaluated retrospectively. Statistical Package for the Social Sciences (SPSS) version 23.0 (IBM, Armonk, NY, USA) was utilized for all statistical analyses.

**Results:**

The median time to progression from the end of initial radiotherapy was 14 months (range, 6–68 months). The most common SRT schedule was 30 Gy (range, 18–50 Gy) in 5 fractions (range, 1–5 fractions). The median follow-up after SRT was 9 months (range, 3–80 months). One-year overall (OS) and progression-free survival (PFS) rates after SRT were 46% and 35%, respectively. Re-irradiation dose and the presence of pseudoprogression were both significant independent positive prognostic factors for both OS (*p* = 0.009 and *p* = 0.04, respectively) and PFS (*p* = 0.008 and *p* = 0.04, respectively). For PFS, progression-free interval > 14 months was also a prognostic factor (*p* = 0.04). The treatment was well tolerated without significant acute toxicity. During follow-up, radiation necrosis was observed in 17 patients (22%), and 14 (82%) of them were asymptomatic.

**Conclusion:**

Hypofractionated SRT is an effective treatment approach for patients with progressive glioblastoma. Younger patients who progressed later than 14 months, received higher SRT doses, and experienced pseudoprogression following SRT had improved survival rates.

## Introduction

Although the first-line management of patients with newly diagnosed glioblastoma has been nearly standardized over the years, the optimal management of progressive disease after initial treatment is still controversial [1]. Repeat surgery, alkylating chemotherapy, bevacizumab, re-irradiation, and clinical trials are commonly recommended treatment modalities [2]. However, there is a paucity of high-quality evidence on the optimal treatment approach for the treatment of progressive disease.

Radiotherapy (RT) is a cornerstone initial treatment approach for almost all patients with newly diagnosed glioblastoma [3]. Therefore, re-irradiation is quite challenging, due to the previous exposure of the organs at risk (OAR) to high-dose radiation. Stereotactic RT (SRT) allows delivery of ablative radiation dose to the target volumes with a sharp dose fall-off, in a few fractions. The improved accuracy of SRT in patient positioning and precise delivery of the dose have led to its widespread use in daily practice. Another fact that might put SRT one step ahead of conventional reirradiation is that RT and temozolomide used in primary treatment induce genetic and phenotypic alterations in tumor cells, resulting in treatment resistance [4]. Hypothetically, an increased dose per fraction might serve as an essential strategy to surmount this resistance.

The literature has a wide range of outcomes with both stereotactic radiosurgery and hypofractionated SRT. Using these techniques, the median survival rates range from 7 to 13 months, 1-year survival rates are reported between 30 and 55%, and neurological toxicity rates range from 5 to 20% [5]. Yet, due to the retrospective and heterogeneous nature of the series, it is challenging to ascertain the optimal dose/fraction schedule, target volume delineation protocol, and subgroups that will benefit most from SRT. Therefore, patient selection is a very relevant issue. There are recommendations for patient selection according to a myriad of prognostic factors. These prognostic factors can be listed as: age, Karnofsky Performance Score (KPS), recurrence interval, tumor volume, surgery before reirradiation, concurrent systemic therapy, reirradiation dose, and O6-methylguanine-DNA methyltransferase (MGMT) gene promoter methylation status [6–12].

We have previously reported our hypofractionated stereotactic re-irradiation results for 37 progressed glioblastoma patients in 2014 [13]. In this current study, we aim to report long-term oncological outcomes, evaluate prognostic factors, and examine the role of SRT as a re-irradiation technique.

## Materials and methods

### Patient cohort

**Fig. 1:**
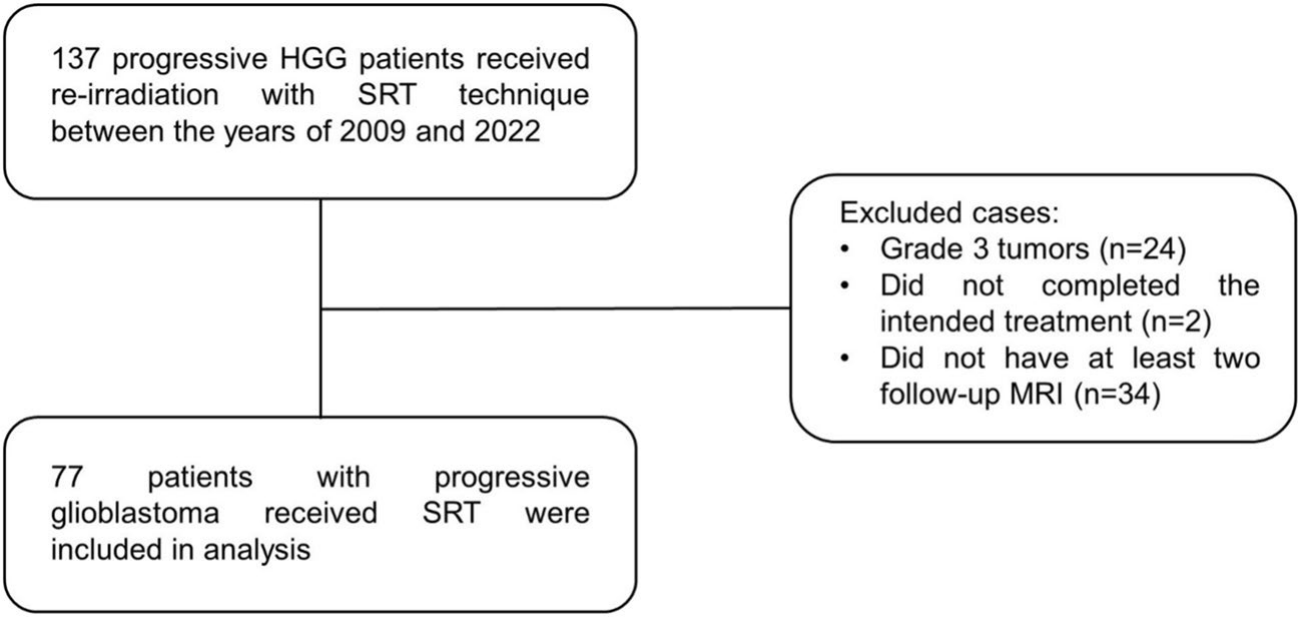
Flow-chart of patient selection. Abbreviations: HGG = high grade glioma, MRI = magnetic resonance imaging, *N* = number, SRT = stereotactic radiotherapy

The medical records of the 77 patients with glioblastoma who received cranial re-irradiation with the hypofractionated SRT (1–5 fractions) in our department between 2009 and 2022 were retrospectively evaluated (Fig. 1). Patients who were older than 18 years of age, had histologically confirmed glioblastoma, completed the planned treatment, KPS of at least 60, and had at least two follow-up scans were included in the study. Since our study covers the period between 2009 and 2022, the World Health Organization (WHO) Classification of Tumors of the Central Nervous System, 4th edition (2007) and updated 4th edition (2016) is used for glioblastoma classification [14, 15]. In our institutional policy, re-irradiation is considered only after six months of initial RT. All patients underwent a tumor resection at the time of the initial diagnosis and received concurrent ± adjuvant temozolomide This study was approved by the institutional ethics board (SBA 23/188, 2023/04–10) and conducted in compliance with the Helsinki Declaration.

### Treatment

**Fig. 2:**
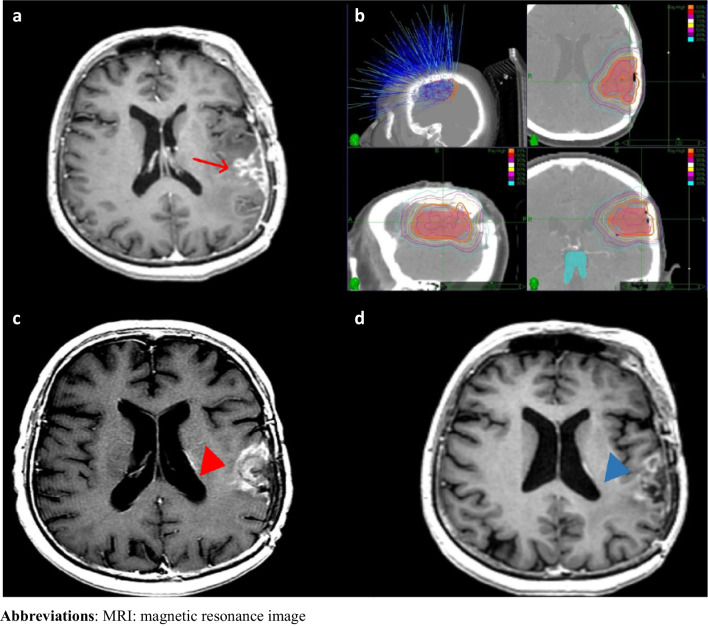
This figure depicts the treatment and follow-up images of a patient diagnosed with recurrent glioblastoma **a.** T1 post-contrast (T1c +) sequence of the MRI for reirradiation planning (red arrow: contrast enhanced recurrent lesion at left temporoparietal lobe) **b.** Treatment plan (dose is prescribed to the 83% isodose line, orange isodose line represents 83% isodose) **c.** At 6 months, MRI showed an increase in contrast-enhancing lesion (red arrowhead) on axial T1C + sequence, pseudoprogression and progression were included in the differential diagnosis **d.** At 8 months after reirradiation, MRI revealed a decrease in contrast-enhancing lesion (blue arrowhead), confirming the presence of pseudoprogression. Abbreviations: MRI: magnetic resonance image

All treatment decisions are made case-by-case on our institutional neuro-oncology tumor board. Patients were immobilized with a thermoplastic mask during the SRT simulation. Computed tomography (CT) and MRI studies with 1.25 mm of slice thickness were performed in the treatment position, at most one week before the initiation of SRT. CT and MRI images were fused for target volume delineation. For patients without a surgical intervention, gross tumor volume (GTV) was delineated as a macroscopic tumor according to the T1 post-contrast (T1c +) sequence of the MRI and clinical target volume (CTV) was not delineated. For patients with a tumor resection at the time of progression, GTV was delineated as a residual tumor if it was present, and CTV was delineated as a resection cavity ± residual tumor according to the T1c + sequence of the MRI. The planning target volume (PTV) was generated with 3 mm of margins to the GTV or CTV. All patients received SRT every other day. Elekta Versa HD linear accelerator (Elekta AB, Stockholm, Sweden), Novalis Brainlab (Chicago, IL, USA), or CyberKnife (Accuray Incorporated, Sunnyvale, CA, USA) were used for treatment delivery (Fig. 2).

We used the linear quadratic formula (EQD2 = nd [(d + α/β)/(2 + α/β)] and biologically effective dose [BED10] = nd [1 + (d/α/β)], n represents the number of fractions and d is the dose per fraction) for calculating the total equivalent dose in 2 Gy per fraction (EQD2) using the ratio of α/β = 10 Gy for the tumor and α/β = 3 Gy for the organs at risk OARs.

### Follow-up

Patients underwent their initial follow-up 4 to 6 weeks after the completion of SRT and those that showed no signs of progression were followed up every 3 months. Patients with suspected radionecrosis or pseudoprogression had follow-up imaging after one to two months to assess for rapid interval changes and guide further management decisions. All suspicious progressions were confirmed with advanced MRI studies such as spectroscopy, diffusion, and perfusion images. Pseudoprogression was defined as an improvement or stabilization of radiographic findings, initially thought to represent a tumor progression, on a constant or decreasing steroid dose within 6 months after the completion of any treatment (SRT or chemotherapy) (Fig. 2). Radionecrosis is defined as a contrast-enhancing lesion with central necrosis and reactive edema in the vicinity of the irradiation field on an MRI at any point during the follow-up beyond 6 months. Response Assessment in Neuro-Oncology (RANO) and modified RANO (mRANO) criteria were used in both the initial progression and the progression after the reirradiation decision [16, 17]. Common Terminology Criteria for Adverse Events (CTCAE) v4.0 was used for toxicity assessment.

### Statistical analysis

Statistical Package for the Social Sciences (SPSS) version 23.0 (IBM, Armonk, NY, USA) was utilized for all statistical analyses, including descriptive statistics, overall survival (OS), and progression-free survival (PFS). All time-related events were defined from the first day of the SRT to the last follow-up, death, or recurrence, whichever came first. Kaplan-Meier method was used for survival analysis and the log-rank test for comparison. Possible prognostic factors were included in the univariate analysis (UVA). *p* < 0.05 was considered statistically significant. The Cox proportional hazards model was used for multivariate analysis (MVA) and variables with potential significance (*p* < 0.1) as a result of UVA were subjected to MVA.

## Results

### Patient, tumor and treatment characteristics

The baseline patient, tumor, and treatment characteristics are summarized in Table 1. At the initial surgery, 43 patients (55.8%) had subtotal resection (STR), whereas 34 patients (44.2%) underwent gross total resection (GRT). Seventy-one patients (92.2%) had IDH wild type, whereas 6 (7.8%) had IDH mutant tumor. Seventy-two patients (93.5%) underwent 6–12 cycles of adjuvant temozolomide. The median time between the end of initial RT and detection of progression was 14 months (range, 6–68 months). All progressions were in the field of initial RT. Repeated resection was performed at the time of progression in 30 patients (39%), and 15 of them (50%) had a GTR. The most common SRT schedule was 30 Gy (range, 18–50 Gy) in 5 fractions (range, 1–5 fractions). The median EQD2 for SRT was 40 Gy (range, 31.2–83.3 Gy). Eighteen patients received concurrent chemotherapy with SRT. The median cumulative brain dose was 100 Gy (range, 72.6–143.3). After SRT, 33 patients received irinotecan + bevacizumab, 13 patients received temozolomide, and 5 patients received nivolumab or pembrolizumab at a median of 6 cycles (range, 2–34 cycles). The first response evaluation was performed after SRT at the median third month (range, 1–6 months). The rates of complete response, partial response, stable disease, and progression rates were 7%, 57%, 21%, and 15%, respectively. The median follow-up after SRT was 9 months (range, 3–80 months). After SRT, 17 (22%) patients experienced a pseudoprogression. The median time to detection of pseudoprogression after SRT was 3.8 months (range, 1–12 months). Local recurrence developed in 24 (31.2%) of the patients who underwent reirradiation. Three of these patients underwent the 3rd course of reirradiation.

**Table 1:** Patient, tumor and treatment characteristics

Characteristics	Number (n, %)
Age (median)	48 y (range, 19–77 y)
Gender	
Male Female	45 (58) 32 (42)
Initial RT dose / fraction	
60 Gy in 30 fractions 40 Gy in 15 fractions	71 (92) 6 (8)
Time to progression (median)	14 months (range, 6–68 months)
Surgery at progression	
Present Absent	30 (39) 41 (61)
Type of surgery at progression	
GTR STR	15 (50) 15 (50)
GTV or CTV volume (median)	36 cc (range, 8–98.5 cc)
SRT dose (median)	30 Gy (range, 18–50 Gy)
SRT EQD2 (median)	40 Gy (range, 31.2–83.3 Gy)
Number of SRT fractions (median)	5 (range, 1–5)
Concurrent CHT	
Present Absent	18 (23) 59 (67)
Type of concurrent CHT	
Temozolomide Irinotecan + Bevacizumab	14 (78) 4 (22)
Pseudoprogression after SRT	
Present Absent	17 (22) 60 (78)
Radionecrosis after SRT	
Present Absent	17 (22) 60 (78)

*CHT* chemotherapy, *CTV* clinical target volume, *GTR* gross tumor resection, *RT* radiotherapy, *SRT* stereotactic radiotherapy, *STR* subtotal tumor resection, *y* years

### Survival outcomes and prognostic factors

One-year OS and PFS rates after SRT were 46% and 35%, respectively. The median OS was 10.1 months (Standard Error [SE]: 1.935; %95 Confidence Interval [CI]: 6.327–13.912) and the median PFS was 8.2 months (SE: 0.754; 95% CI: 6.722–9.678). Six patients diagnosed with IDH mutant grade 4 glioma were also included in the study because they met the definition of glioblastoma at the time. However, only grade 4 IDH wild-type tumors are classified as glioblastoma in the most recent WHO (WHO CNS5) classification [18]. Accordingly, for IDH wild-type patients, one-year OS and PFS rates were 43% and 34%, respectively. The median OS was 8.6 months (SE: 1.549; 95% CI: 5.635–11.705), and the median PFS was 8.1 months (SE: 0.637; 95% CI: 6.899–9.397). There was no statistically significant difference in OS and PFS between patients with IDH mutant and wild type tumors (*p* = 0.7 and *p* = 0.2, respectively).

Prognostic factors associated with increased OS and PFS are listed in Table 2 and 3. Age (≤ 60 vs. > 60), gender (male vs. female), progression time (≤ 14 months vs. > 14 months), SRT dose (< 40 Gy vs. ≥ 40 Gy), pseudoprogression after SRT (present vs. absent) were parameters affecting OS and PFS in univariate analyses. In multivariate analysis, EQD2 of SRT ≥ 40 Gy and presence of pseudoprogression were significant independent positive prognostic factors for both OS and PFS; progression-free interval > 14 months was also a significant variable affecting PFS (Table 3).

**Table 2:** Univariate analysis for overall, and progression free survival

Variables	1-y OS (%)	p	1-y PFS (%)	p
Age		***0.02***		***0.03***
≤ 60 y (*n* = 61) > 60 y (*n* = 16)	48 33		39 20	
Gender		***0.009***		***0.025***
Male (*n* = 45) Female (*n* = 32)	38 57		30 44	
Progression time		***0.01***		***0.007***
≤ 14 m (*n* = 37) > 14 m (*n* = 40)	32 60		22 47	
Repeated surgery		0.42		0.467
Present (*n* = 30) Absent (*n* = 47)	54 40		38 34	
Concurrent CHT with SRT		0.63		0.828
Present (*n* = 18) Absent (*n* = 59)	56 42		44 32	
Dose of SRT (EQD2)		***0.001***		***0.001***
< 40 Gy (*n* = 30) ≥ 40 Gy (*n* = 47)	20 56		15 47	
Volume of GTV or CTV		0.169		0.172
≤ 34 cc (*n* = 40) > 34 cc (*n* = 37)	53 37		40 30	
Pseudoprogression after SRT		***0.048***		***0.005***
Present (*n* = 17) Absent (*n* = 60)	75 37		59 28	

*CHT* chemotherapy, *CTV* clinical target volume, *EQD2* Equivalent dose in 2 Gy per fraction, *m* months, *n* number, *OS* overall survival, *PFS* progression-free survival, *SRT* stereotactic radiotherapy, *y* years

**Table 3:** Results of multivariate analysis for overall, and progression-free survival

Survival	Variables	HR	95% CI	*p*
	≤ 60 y > 60 y	1 0.5	0.27–1.025	0.059
	Female Male	1 1.7	0.982–2.951	0.058
OS	Progression at > 14 months Progression at ≤ 14 months	1 1.6	0.939–2.792	0.083
	SRT ≥ 40 Gy (EQD2) SRT < 40 Gy (EQD2)	1 2.2	1.223–3.996	***0.009***
	Pseudoprogression present Pseudoprogression absent	1 1.8	1.015–3.543	***0.045***
	≤ 60 y > 60 y	1 0.6	0.323–1.225	0.17
	Female Male	1 1.4	0.87–2.535	0.14
PFS	Progression at > 14 months Progression at ≤ 14 months	1 1.7	1.019–3.086	***0.04***
	SRT ≥ 40 Gy (EQD2) SRT < 40 Gy (EQD2)	1 2.1	1.221–3.695	***0.008***
	Pseudoprogression present Pseudoprogression absent	1 1.8	1.008–3.420	***0.04***

*CI* confidence interval, *EQD2* equivalent dose in 2 Gy per fraction, *HR* hazard ratio, *OS* overall survival, *PFS* progression-free survival, *SRT* stereotactic radiotherapy

### Toxicity

Treatment was well-tolerated without any ≥ grade 3 acute toxicity. During follow-up, radiation necrosis was observed in 17 patients (22%) and 14 (82%) of them were asymptomatic. The median cumulative brain dose of these patients was 100 Gy (range, 91.2–143.3). No significant difference was detected in the median cumulative brain dose, concurrent chemotherapy use during reirradiation, GTV volumes or second-line RT timing between patients with and without radionecrosis (*p* = 0.3, *p* = 0.5, *p* = 0.9, *p* = 0.2 respectively). However, more radionecrosis was observed in patients who were younger at the time of second-course RT, underwent surgery before reirradiation, and had pseudoprogression (*p* = 0.04, *p* = 0.03, *p* < 0.01, respectively). Upon careful examination of the three patients suffering from symptomatic radionecrosis, all three had a cumulative brain dose of 100 Gy, but two used temozolomide concurrently with SRT followed by irinotecan + bevacizumab. Patients with symptomatic radiation necrosis were managed by steroids and/or bevacizumab. None of the patients experienced any other ≥ grade 3 late toxicity.

## Discussion

For patients diagnosed with glioblastoma, progression is inevitable and in case of progression, the prognosis is dismal. In our cohort, which has one of the highest number of patients treated with hypofractionated SRT in the literature, we present our outcomes over a 12-year period. The median OS with SRT was 10.1 months, and the median PFS was 8.2 months. These results support the existing evidence in the literature, indicating that hypofractionated SRT is a viable therapeutic option for patients with recurrent glioblastoma with low symptomatic radionecrosis rates and toxicity. Our findings indicate that certain parameters have a substantial favorable impact on survival and might be used in patient selection. These factors include a longer progression-free interval after initial treatment, ≥ 40 Gy EQD2 of re-irradiation, and the presence of pseudoprogression after SRT. Also, patients aged 60 and over had a poorer prognosis, which was not statistically significant but could be clinically relevant (*p* = 0.05).

After the decision for salvage RT is made, the plethora of salvage reirradiation approaches described in the literature make it difficult to choose the most appropriate RT technique and dose/fractionation schedule. In this challenging issue, providing the appropriate treatment strategy and implementing it thoroughly is of utmost significance. In our study, the SRT method effectively addresses this requirement by offering high accuracy and precise treatment. Additionally, we observed a survival benefit in patients receiving doses of 40 Gy and higher. Examining the radiobiology of the recurrent disease, which is believed to be resistant to radiation, appears to be pertinent in this context [4]. Currently, there is a disagreement on recommended doses for reirradiation. Kazmi et al. [19], did not find a dose-response relationship with conventional doses. However, they found that patients treated with brachytherapy had better OS. Furthermore, PFS benefits have been demonstrated with radiosurgery and hypofractionation. This suggests the presence of potential resistance to radiation and implies that a correlation between dose and response may only be observed when the dose is above a certain fraction dose and a certain BED threshold. Fogh et al. [20], demonstrated that there was a tendency for survival to improve as doses reached 35 Gy or higher, with a fraction dose of 3.5 Gy (*p* = 0.07). Chapman et al. [12] established the dose threshold for SRS as 40 Gy BED10 and for non-SRS treatments as 45 Gy BED10. Again, in the literature, dose response relationships have been shown for different dose thresholds, such as 30–35 Gy for hypofractionated RT and 36 Gy and 41.4 Gy for conventional RT [21–24]. In our results, the median dose per fraction was 6 Gy, and patients receiving ≥ 40 Gy of EQD2 had improved survival rates as compared with < 40 Gy, which supports the hypothesis that a certain threshold BED value may be needed to overcome radioresistance and show the desired effect.

Surgical resection is an important salvage treatment option, but resection before reirradiation is a significant subject of debate in the literature. Although resection contributes to a survival rate of 8–9 months in progressive glioblastoma, its place before second-course RT is controversial [25]. While there is no robust data showing the contribution of resection before RT, a significant number of studies show its contribution to PFS and OS [9, 11, 26]. Also, some series evaluate surgery and RT together as a sequential maximal and optimal treatment method [10, 27, 28]. We did not report the contribution of pre-RT resection to PFS and OS; in fact, we showed that there was more radionecrosis in these patients. In our rationale, given the limited treatment options and inevitable risk of disease progression in this patient group, it is prudent to utilize a minimal number of treatments that have the highest efficacy at the time of progression. This approach can expand the available treatment options for future progressions and mitigate the already elevated risk of adverse effects.

Another controversial issue is the use of chemotherapy simultaneously with second-course irradiation. Many retrospective studies failed to demonstrate survival benefits with the addition of concurrent systemic therapy (i.e., temozolomide, bevacizumab, irinotecan, etc.) to the re-irradiation [12, 20]. However, there were also studies that argued the opposite and showed that chemotherapy might potentially enhance the therapeutic outcomes [29–31]. In a secondary analysis of Radiation Therapy Oncology Group (RTOG) 0525, Shi et al. [32], compared treatment modalities in patients diagnosed with progressive glioblastoma and found that median survival times were 4.8, 8.2, 10.6, and 12.2 months for patients who received no treatment, radiation treatment only (SRS, fractionated RT or brachytherapy), systemic therapy, or radiation and systemic therapy, respectively. However, no difference was observed between modalities in survival analyses. In the recently published prospective randomized phase II RTOG1205 trial, 179 patients were randomized to either re-irradiation + bevacizumab or bevacizumab alone [33]. In patients who received re-irradiation + bevacizumab, 6-month PFS was significantly improved as compared with bevacizumab alone (54% vs. 29%, *p* = 0.001). However, the beneficial effects of bevacizumab in concomitant use are not due to increased radiosensitivity, unlike alkylating agents. The primary cause of these effects is often attributed to an augmentation in vascular stability and oxygenation [34]. In our results, 18 of 77 patients received concurrent chemotherapy and SRT, mostly temozolomide (78%), and survival rates were similar as compared with patients who received SRT alone. Ultimately, it is evident that there is still a great need for studies focusing on the subgroup of patients who would benefit most from concurrent systemic therapy and SRT.

The most important concerns about reirradiation are radionecrosis and the potential significant toxicity that may arise with a second course of irradiation. Radionecrosis rates ranging from 0 to 37% have been reported with radiosurgery and hypofractionated SRT series [5]. The radionecrosis rate of 22% in our study aligns with the existing literature and 82% of them were asymptomatic. We observed that there was more radionecrosis in patients who were younger at the time of second-course RT, underwent surgery before RT, and had pseudoprogression. Although no significant difference was detected in concurrent chemotherapy use, GTV volumes, or reirradiation interval, second-line chemotherapy and concurrent chemotherapy were used in 3 patients with symptomatic radionecrosis.

The results of our study demonstrated the good efficacy of SRT, along with the evaluation of significant prognostic factors such as age, progression-free interval, re-irradiation dose, and pseudoprogression. However, there are certain limitations to consider, including the retrospective nature of the study, a limited sample size, a short follow-up period, and uncertainty regarding the MGMT status of the patients. We also couldn't provide the patients' KPS scores. However, in our clinic, patients are expected to have a KPS of at least 60 for the second series of hypofractionated SRT.

## Conclusions

To conclude, we found that hypofractionated SRT is an effective treatment approach for patients with progressive glioblastoma. In our retrospective cohort, patients who had a progression-free interval exceeding 14 months and received a minimum of 40 Gy EQD2 of SRT had improved rates of survival. In addition, pseudoprogression may also occur after SRT, and it is associated with better survival. Prospective studies focusing on the optimal timing, the dose of re-irradiation, prognostic factors, and the pathogenesis underlying the pseudoprogression are clearly needed. Although the prognosis of patients with progressive glioblastoma is still grim, the adoption of molecular markers on a regular basis and the identification of patient subgroups that would benefit most from SRT might potentially enhance oncological outcomes.

## Data Availability

No datasets were generated or analysed during the current study.
